# Peptonate of Mercury

**Published:** 1881-12

**Authors:** 


### PEPTONATE OF MERCURY.

Dr. Martineau (*Progres Medical*) claims that the albuminate of Bamberger often causes local troubles, and advises the following injection: Peptone, mercury bichloride, one-twelfth grain each; distilled water, fifteen minims. This hypodermic medication is to be preferred to medication by the stomach. First: Because its action is more certain. Second: Because it does not derange the digestive organs. Third: Because the exact quantity taken into the system can be easily determined.

